# SRpHi ratiometric pH biosensors for super-resolution microscopy

**DOI:** 10.1038/s41467-017-00606-4

**Published:** 2017-09-18

**Authors:** Douglas S. Richardson, Carola Gregor, Franziska R. Winter, Nicolai T. Urban, Steffen J. Sahl, Katrin I. Willig, Stefan W. Hell

**Affiliations:** 1 Department of NanoBiophotonics, Max Planck Institute for Biophysical Chemistry, 37077 Göttingen, Germany; 2 Department of Molecular and Cellular Biology, Harvard Center for Biological Imaging, Harvard University, Cambridge, MA 02138 USA; 3 Nanoscale Microscopy and Molecular Physiology of the Brain, University Medical Center Göttingen, 37075 Göttingen, Germany; 4 Department of Optical Nanoscopy, Max Planck Institute for Medical Research, 69120 Heidelberg, Germany

## Abstract

Fluorescence-based biosensors have become essential tools for modern biology, allowing real-time monitoring of biological processes within living cells. Intracellular fluorescent pH probes comprise one of the most widely used families of biosensors in microscopy. One key application of pH probes has been to monitor the acidification of vesicles during endocytosis, an essential function that aids in cargo sorting and degradation. Prior to the development of super-resolution fluorescence microscopy (nanoscopy), investigation of endosomal dynamics in live cells remained difficult as these structures lie at or below the ~250 nm diffraction limit of light microscopy. Therefore, to aid in investigations of pH dynamics during endocytosis at the nanoscale, we have specifically designed a family of ratiometric endosomal pH probes for use in live-cell STED nanoscopy.

## Abstract (web summary)

Ratiometric fluorescent pH probes are useful tools to monitor acidification of vesicles during endocytosis, but the size of vesicles is below the diffraction limit. Here the authors develop a family of ratiometric pH sensors for use in STED super-resolution microscopy, and optimize their delivery to endosomes.

## Introduction

Endocytosis is an essential cellular process that directs the internalization and intracellular trafficking of membrane proteins, their ligands, and other soluble molecules throughout the cell^1^. An important mechanism that allows the endocytic machinery to carry out its functions is the gradual acidification of endosomes as they mature from nascent membrane-derived vesicles (pH ~7.4) to lysosomes (pH < 5)^2^. Therefore, endosomal pH sensors have been essential tools of many researchers investigating intracellular trafficking events within the cell^3–5^. Unfortunately, the small size of endosomes has hindered their investigation in live-cell microscopy studies. Many structures in the endocytic pathway are <250 nm in size, placing them below the diffraction limit of conventional light microscopes and rendering it impossible to distinguish close-lying structures such as densely packed endosomes or fine endosomal projections known as tubules^6^. Often, researchers use genetically modified model systems with enlarged endosomes to better visualize these structures^7^. Recent advances in light microscopy now allow imaging of cells at sub-diffraction resolutions of 50 nm or better^8^.

Although a number of fluorescent pH probes exist (Table 1 and refs. ^3–5^), most have characteristics that make them difficult or impossible to use for pH measurements in endosomes. For example, some do not target the endocytic pathway and simply diffuse throughout the cytoplasm. Others contain only a single fluorophore that changes in fluorescence intensity. Once this fluorescence is quenched, the probe or structure it resides in is no longer visible. Ratiometric probes that utilize two fluorophores may occupy a wide range of the visible spectrum (for example, 500–700 nm for an EGFP/mCherry-based probe) that is detectable by a fluorescence microscope, thereby reducing the remaining spectrum available for co-labeling of other molecules of interest. Some pH sensors are based on chromophores with low brightness (lysosensor, SNARF) and high bleaching rates in aqueous environments (fluorescein) or are not compatible with the higher demands of super-resolution microscopy. Finally, many have a rather limited dynamic range, or a near-neutral p*K*_a_ value that prevents the use of their full dynamic range in acidic vesicles, making it difficult to detect modest changes in pH. Here, we present a family of endosomal pH sensors that are developed specifically for STED nanoscopy, namely, the super-resolution pH indicators (SRpHi). STED was the super-resolution method of choice as it acquires raw data with no need for later computational analysis that would complicate and possibly render data analysis (such as ratio calculations) impossible.

**Table 1:** Properties of biological pH sensors

pH Probe	Fluorophore #1	Fluorophore #2	Ratiometric	QY/EC/Brightness Fluorophore #1	QY/EC/Brightness Fluorophore #2	pKa	Usable pH range	Comments	Ref
*Non-ratiometric single fluorophore*									
FITC	FITC		No	0.93/75,000/69.7		6.1	5.0–9.0	Rapid bleaching in aqueous buffer. Protein conjugation decreases fluorescence. No fluorescence at low pH. Primarily a cytosolic pH probe.	^23^
pHrodo	pHrodo		No	0.34/65,000/22.1		6.5	4.0–8.0	No fluorescence at neutral/basic pH	^24^
pHluorin	Mutant GFP		No	0.08/41,000/3.3		7.1	5.5–7.0	No fluorescence at low pH.	^25^
sepHluorin	EGFP mutant		No	NA/NA/22		7.2	6.0–7.5	No fluorescence at low pH.	^25^
pHuji	mApple mutant		No	0.22/31,000/6.8		7.7	6.5–8.5	No fluorescence at low pH.	^26^
*Ratiometric single fluorophore*									
Ratiometric pHluorin	GFP2 mutant		Yes, dual excitation	NA		NA	6.0–7.5		^27^
BCECF	BCECF		Yes, dual excitation	0.84/90,000/75.6		7.0	6.0–8.0	Primarily a cytosolic pH probe.	^4^
SNARF-4F	SNARF-4F		Yes, dual excitation & emission	0.09/47,100/4.2		6.4	6.8–7.4	Low fluorescence at neutral pH	^28^
LysoSensor yellow/blue	LysoSensor yellow/blue		Yes	0.34/23,000/7.8		4.2	3.5–6.0		^29^
*Ratiometric dual fluorophore*									
mCherry-pHluorin	sepHluorin	mCherry	Yes	NA/NA/22	0.22/72,000/15.8	7.1	5.5–7.0		^9^
SRpHi1	EYFP	STAR410	Yes, dual excitation	0.61/83,600/51.0	0.44/14,000/6.1	6.9	5.0–7.0		
SRpHi2	EGFP	STAR512	Yes, dual excitation	0.60/56,000/33.6	0.82/92,000/75.4	5.9	4.5–6.5		
SRpHi3	sepHluorin	STAR512	Yes, dual excitation	NA/NA/22.0	0.82/92,000/75.4	7.1	5.5–7.5		
SRpHi4	EYFP	STAR400	Yes, dual excitation	0.61/83,600/51.0	0.86/20,900/18.0	6.9	5.0–7.0		

Brightness in units of mM^−1^·cm^−1^; EC, extinction coefficient (M^−1^·cm^−1^); QY, quantum yield.

The design focuses on three key factors. Due to the need for high-speed scanning (to capture endosome dynamics in living cells) and thus a need for high-fluorescence flux from these structures (due to short pixel dwell times), we focus on pH-sensitive fluorophores that are efficient in absorbing excitation light (high extinction coefficient), converting excitation light to fluorescence emission (high quantum yield), and are photostable. All pH-sensitive fluorophores used here have a brightness value between 22 and 51 (Table 1). Secondly, we develop an efficient delivery method that functions across many cell types to rapidly load pH sensors into endosomes while limiting their diffusion/attachment to other regions of the cell. Thirdly, sensors are designed to require a single STED laser, occupy a narrow region of a fluorescent microscope’s detection band, and maintain a large dynamic range over relevant pH values.

To best address these parameters, the SRpHi molecules are designed as ratiometric pH probes based on the historical two-fluorophore design in which one fluorophore displays a relatively higher degree of pH-dependent fluorescence quenching than the other^9, 10^. All of the fluorophores utilized here have been previously characterized for use in STED microscopy and are known to be sufficiently bright and photostable (Table 1 and refs.^11–13^).

## Results

**Fig. 1:**
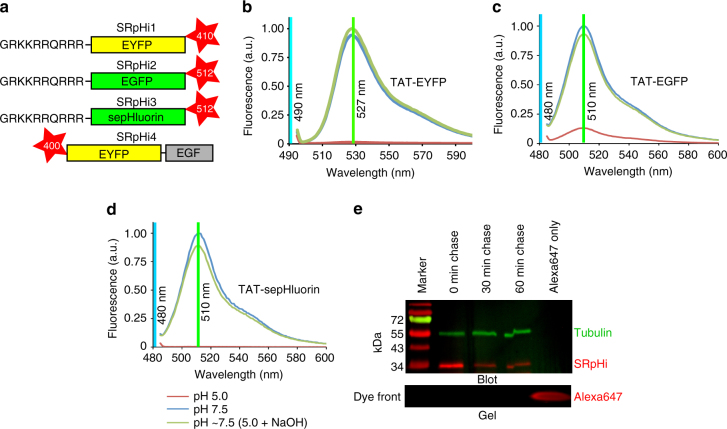
SRpHi is a reversible and stable pH biosensor. **a** Diagrammatic representation of the SRpHi structure. SRpHi molecules consist of a fluorescent protein backbone genetically fused to the cell-penetrating peptide TAT or epidermal growth factor (EGF). In addition, organic dye molecules are covalently attached via an amide linkage in a ~2:1 ratio. **b**–**d** Normalized fluorescence emission spectra of TAT-EYFP **b**, TAT-EGFP **c**, or TAT-sepHluorin **d** were obtained from purified protein dissolved in citric acid/phosphate buffer of pH 7.5 (*blue line*) or 5.0 (*red line*). Spectra were then reacquired after addition of concentrated NaOH to return the pH 5.0 solution to ~pH 7.5 (*green line*). The wavelength of excitation light used for each experiment, and the fluorescence maximum of each fluorophore is indicated. **e** Pulse-chase experiments were performed using an EGFP-based SRpHi molecule conjugated to the far-red dye Alexa 647 (to prevent acquisition of signal arising from non-denatured EGFP). Lysates from MEF cells undergoing the indicated treatments were separated by SDS-PAGE and run alongside a molecular weight marker and free Alexa 647 dye. The dye-front of the gel was analyzed for far-red fluorescence (>650 nm, *red*, *bottom panel*) prior to transfer to PVDF membrane and immunolabelling (*top panel*). Primary antibodies against gamma-tubulin were visualized by reaction with secondary antibodies conjugated to Cy3 (*green*) and used as a loading control. SRpHi was visualized in the far-red spectrum, as the denatured protein did not emit fluorescence

The basic construction of a SRpHi molecule is presented in Fig. 1a. In this design, a fluorescent protein with reversible, acid-sensitive fluorescence quenching (Fig. 1b–d) is genetically fused to the positively charged 10 amino acid cell-penetrating peptide sequence from HIV-1 Tat (GRKKRRQRRR)^14^. TAT-fused fluorescent proteins were purified from bacteria (Supplementary Fig. 1a) and labeled with either Abberior STAR400, STAR410, or STAR512 dye (Supplementary Fig. 1b). Table 1 contains additional details on the composition of the probes—SRpHi1, SRpHi2, SRpHi3, and SRpHi4—presented here.

### SRpHi targets to the endosomal pathway

**Fig. 2:**
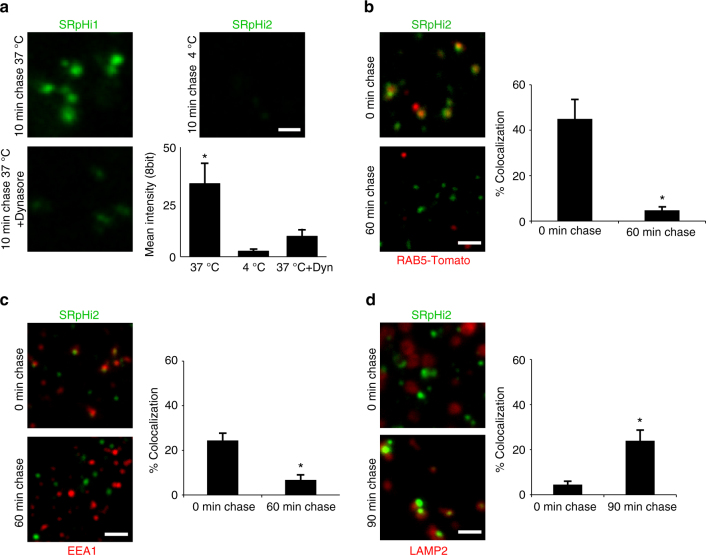
SRpHi is targeted to the endocytic pathway. **a** HeLa cells were untreated (*top images*) or incubated with 80 μM Dynasore (*bottom image*) for 45 min. Cells were then pulsed with SRpHi1 for 10 min at 37 °C (*left images*) or 4 °C (*right image*) followed by fixation. The mean 8-bit intensity of all detectable endosomes was calculated and displayed. **b**–**d** CV-1 cells transfected with tdTomato-RAB5a **b** or untransfected **c**, **d** were pulsed with SRpHi2 for 10 min followed by fixation or a 60–90 min chase in normal growth medium. Cells were immunostained for EEA1 **c** or LAMP2 **d**, and colocalization between SRpHi and RAB5a **b**, EEA1 **c**, or LAMP2 **d** was determined using a custom-written Matlab script. Mean percent colocalization from 5 to 10 cells was calculated and plotted for the pulse and pulse/chase conditions. *Error bars* represent standard deviation from the mean; * represents a *p*-value of <0.05 as calculated by a 2-tailed Student’s *t*-test. Data are representative of three independent experiments. *Scale bars* = 1 µm

A pulse/chase experiment was performed to confirm the intracellular stability of SRpHi. Although a decrease in SRpHi protein was detected between the initial 10 min pulse and a chase of 30 min, no further decrease was seen after a 60 min chase (Fig. 1e). Additionally, no accumulation of free dye that would indicate SRpHi protein breakdown was detected (Fig. 1e). Therefore, we attribute the initial decrease in SRpHi protein levels between the pulse and 30 min chase to the loss of membrane-bound but not internalized SRpHi during washing and fixation, and the possible recycling of SRpHi to the extracellular space. Together, these data indicate that the SRpHi probe is stable intracellularly for at least 60 min post-internalization. In order to determine if the TAT sequence, which interacts with the negatively charged plasma membrane, correctly targeted the SRpHi probe to the endocytic pathway, uptake of SRpHi was monitored in fixed mouse embryonic fibroblast (MEF) and HeLa cells. Prior to fixation, HeLa cells were incubated with SRpHi for 10 min at 37 °C, 4 °C, or at 37 °C after the cells were pre-treated with 80 µM Dynasore for 45 min. Figure 2a displays uptake of SRpHi into small, vesicle-like structures. This uptake could be reduced by inhibiting endocytosis by cooling the cells or through pharmacological methods (using the dynamin inhibitor Dynasore). The probe-containing vesicles were shown to rapidly colocalize with transiently expressed tdTomato-RAB5a (Fig. 2b) and endogenous EEA1 (Fig. 2c), which are both commonly used markers of early endosomes, after 10 min of continuous uptake. A significant decrease in colocalization between SRpHi and tdTomato-RAB5a or EEA1 was noted when the initial 10 min incubation (pulse) was followed by a 60 min incubation at 37 °C in SRpHi-free growth medium (chase, Fig. 2b, c). Further, we observed a significant increase in SRpHi colocalization after a 90 min chase interval with the late endosome/lysosome marker LAMP2 in a similar pulse/chase experiment (Fig. 2d). These results confirm that SRpHi is efficiently targeted to early and late endosomes, prior to delivery to the lysosomal compartment.

### SRpHi probes are compatible with STED microscopy

**Fig. 3:**
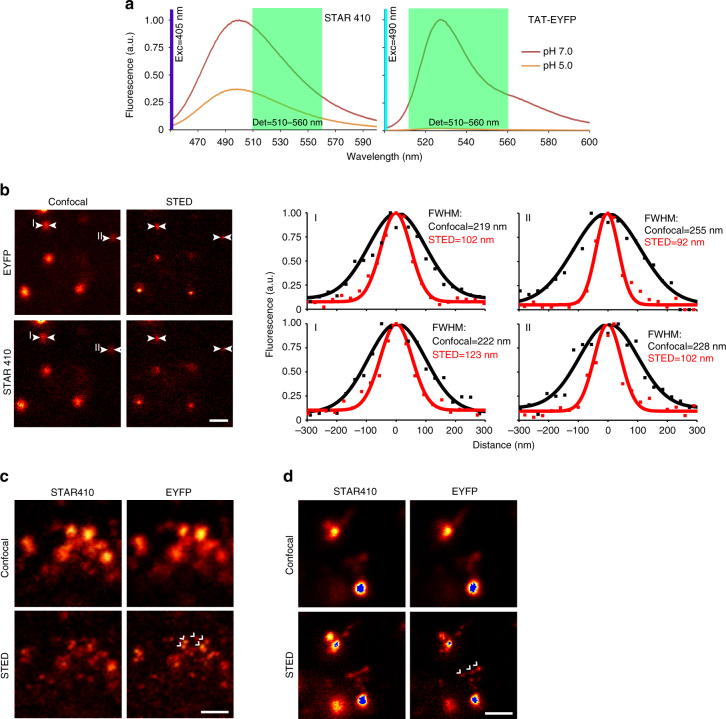
SRpHi is a super-resolution compatible biosensor. **a** Relative fluorescence of STAR410 and TAT-EYFP at pH 7.0 and 5.0 is determined by fluorescence spectroscopy. The detection range of our custom-built microscope is indicated by *light green shading*. STAR410 and EYFP were excited by 405 and 490 nm light, respectively. **b** MEF cells were pulsed with SRpHi1 for 10 min and imaged in PBS using a custom-built STED microscope. Full width at half maximum (FWHM) calculations are presented for two individual endosomes imaged in both confocal and STED mode. **c** CV-1 cells were loaded with SRpHi1 for 10 min, washed, and imaged. Clusters of endosomes smaller than the diffraction limit **c** and tubule-like structures protruding from early endosomes **d** were imaged. Structures only visible by STED are highlighted by *white arrows*. *Blue pixels* in **d** represent saturation. Saturation of these pixels was required to be able to see the much dimmer tubule-like structures. *Scale bars*: 600 nm **b**, 250 nm **c**, **d**

Our initial probe, SRpHi1, was comprised of the Abberior STAR410 dye and the fluorescent protein EYFP (Fig. 1a; Table 1). These fluorophores were first analyzed in solution to determine their effectiveness as indicators of pH. Figure 3a displays fluorescence emission spectra of STAR410 and TAT-EYFP in solution at pH 7.0 and 5.0. At pH 5.0, EYFP is reduced to ~2% of its brightness at pH 7.0. In comparison, STAR410 is only reduced to ~37%. Comparison of the area under each curve (integral) across the detection range of our microscope suggested a potential ratiometric intensity change (dynamic range) of nearly 10-fold.

However, as differences in excitation wavelength and intensity, the detection range and intracellular environment can all affect biosensor performance, we moved to a live-cell model system in order to fully characterize SRpHi. Live, intact MEF cells were imaged using a custom-built STED microscope that has been previously described^11, 15^. Two excitation lasers, at 405 and 490 nm, were used to excite the STAR410 and EYFP fluorophores sequentially. A pulsed STED beam at 590 nm was used for stimulated emission depletion (fluorescence silencing) of both fluorophores. This setup produced excellent separation of both dyes with only a minor (<10%) bleed-through of the EYFP signal into the STAR410 channel (Supplementary Fig. 2). Due to the relative intensities of the fluorophores, this bleed-through was negligible and spectral unmixing resulted in no noticeable improvement to either image (data not shown). Figure 3b displays confocal and STED images of MEF cells loaded with SRpHi1 for 10 min and imaged at room temperature in phosphate-buffered saline (PBS). As expected, a quantifiable resolution increase was observed for both the STAR410 and EYFP channels. Although not all endosomes were below the diffraction limit in size, STED imaging revealed a population of endosomes <100 nm in diameter (examples in Fig. 3b–d). This was a significant resolution improvement over diffraction-limited confocal imaging where endosomes appeared as objects more than 200 nm in size (Fig. 3b–d). STED microscopy proved particularly useful for resolving clusters of tightly packed endosomes (Fig. 3c) and tubular structures protruding from vesicles (Fig. 3d) that were not resolved by traditional confocal microscopy.

### In vivo characterization of SRpHi

**Fig. 4:**
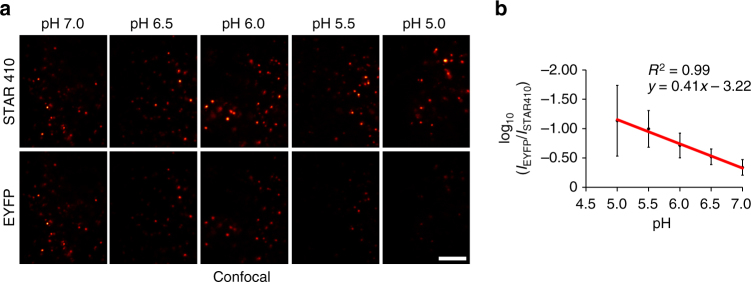
SRpHi probes respond to pH changes in vivo. **a** MEF cells pulsed with SRpHi1 for 10 min were equilibrated to the indicated pH by application of an external citric acid/phosphate buffer supplemented with 140 mM KCl and 1 µM nigericin. Cells were imaged in confocal mode. EYFP images have been normalized to the corresponding STAR410 image for visualization. *Scale bar*: 5 µm. **b** Cells were imaged as in **a** with STED. A mask of all endosomes was created and the ratio of EYFP/STAR410 signal was calculated for each endosome (*n* > 150 endosomes for each pH value), averaged for each pH value, and their log_10_ value plotted. *Error bars* represent standard deviation from the mean. Log_10_ EYFP/STAR410 values were fit to a linear equation (*R* ^2^ = 0.99)

**Fig. 5:**
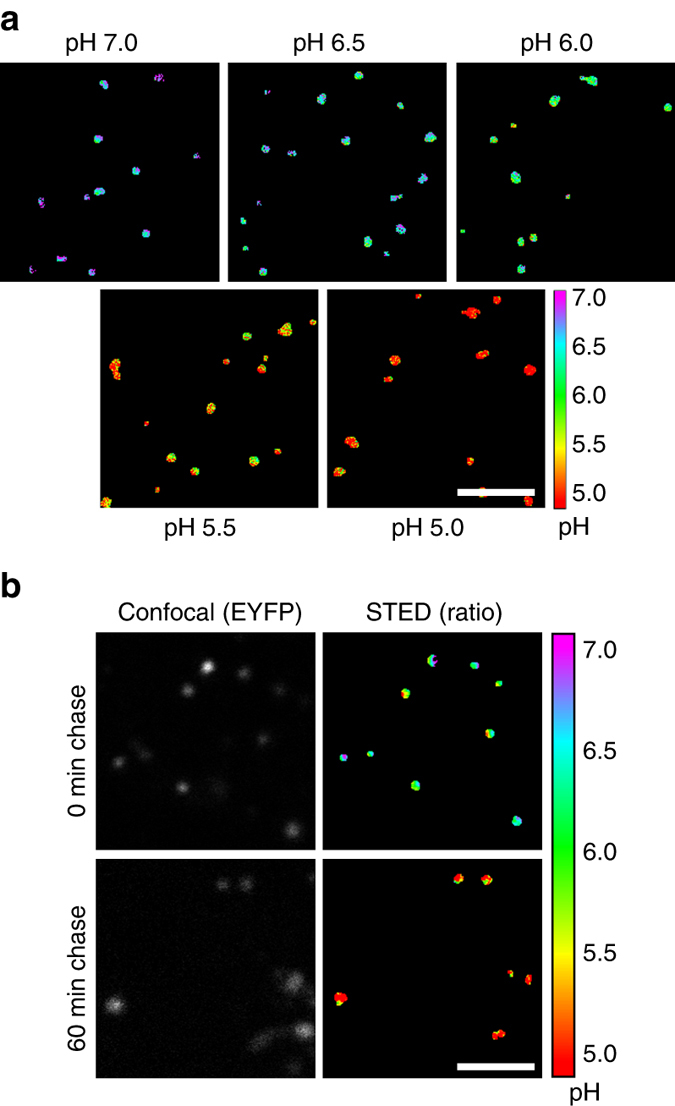
Ratiometric imaging of SRpHi produces reliable measurements of endosomal pH. **a** Ratiometric (FP/dye) STED images of live MEF cells loaded with SRpHi1 and equilibrated to the indicated pH using a citric acid/phosphate buffer supplemented with 140 mM KCl and 1 µM nigericin. **b** Ratiometric STED imaging of pulse-chase experiments performed in MEF cells loaded with SRpHi1. Cells in the *top row* received a 10 min pulse of SRpHi1 prior to imaging. Cells in the *bottom row* received the same pulse followed by a 60 min chase in SRpHi1-free medium. *Scale bars*: 2 µm

In order to compare the in vivo performance of SRpHi1 to our initial characterizations in solution, MEF cells were loaded with SRpHi1 for 10 min followed by incubation in a citric acid/phosphate buffer adjusted to various pH values and supplemented with nigericin and KCl (140 mM). This is an established method to equilibrate intracellular pH to an extracellular buffer^16^. As expected, an obvious decrease in EYFP fluorescence relative to STAR410 fluorescence was seen as the intracellular pH was reduced (Fig. 4a). We further determined the average signal intensity in each channel for over 150 endosomes at each of the following pH values: 7.0, 6.5, 6.0, 5.5, and 5.0. These data were used to produce the ratiometric standard curve presented in Fig. 4b. The log_10_ of the ratiometric data fit well to a linear function (*R* ^2^ = 0.99), and resulted in a greater than six-fold change over the pH range tested. This is greater than the many common ratiometric pH probes currently available (2–5-fold)^3–5^. In addition, the high acid stability of the STAR410 dye ensures endosomes remain visible over the full range of endosomal pH values unlike single-dye pH sensors. A higher dynamic range is possible, however the 405 nm laser power was specifically kept at low levels to prevent excitation of EYFP by the 405 nm light, and to prevent photobleaching and/or photodamage to the cells. Figure 5a displays ratiometric images of endosomes equilibrated to various pHs (5.0–7.0). Additionally, a pulse/chase experiment further confirmed the accuracy of the standard curves and SRpHi’s ability to produce live-cell super-resolution ratiometric images. In the experiment, SRpHi correctly reported the decrease in endosomal pH from 10 to 60 min after internalization (Fig. 5b).

### SRpHi2 and 3 have increased dynamic range

Based on our original design, we produced additional pH probes with enhancements to serve a broader range of endocytosis experiments. SRpHi2 and 3 incorporated changes to enhance their dynamic range, shift their excitation spectra away from the near UV, and/or alter their optimal pH range. Both probes incorporated the highly acid-stable STAR512 dye that maintained performance at pH values below 5.0 when paired with EGFP in SRpHi2. Analysis of these probes was performed by modifying a previously described STED microscope for two-color excitation and detection^17^ (Supplementary Fig. 3). Measurements in solution and intact cells showed that SRpHi2 has a dynamic range of more than 15-fold over a pH range of 6.5–4.5 during STED imaging (Supplementary Fig. 4a–c). A similar analysis of SRpHi3 determined a dynamic range of over 20-fold from pH 7.5 to 5.5 (Supplementary Fig. 4d–f). This suggests that very large dynamic ranges are attainable in live cells at STED resolution, far exceeding the current complement of available pH probes. In addition, SRpHi1-3 display unique optimal pH ranges, lending themselves to the specific analysis of different stages of endocytosis (Supplementary Table 1).

### SRpHi fusions target probes to specific endosomal pathways

**Fig. 6:**
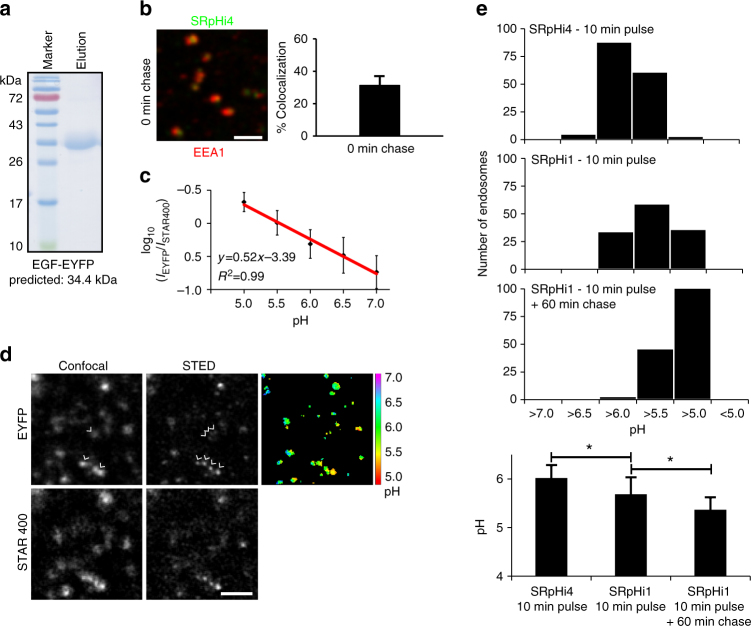
SRpHi probes can be fused to endogenous ligands. **a** The identity and purity of SRpHi4 was confirmed by SDS-PAGE and Coomassie stain. SRpHi4 consists of a C-terminal fusion of mature human EGF to EYFP. Additionally, SRpHi4 is conjugated to the STAR400 dye. **b** SRpHi4 (*green*) was loaded into CV-1 cells for 10 min followed by fixation and immunostaining against the early endosomal marker EEA1 (*red*). Colocalization between SRpHi4 and EEA1 was calculated as in Fig. 2. **c** CV-1 cells pulsed with SRpHi4 for 10 min were equilibrated to the indicated pH by application of an external citric acid/phosphate buffer supplemented with 140 mM KCl and 1 µM nigericin. Two-channel (STAR400 and EYFP) confocal and STED imaging was performed. The STED STAR400 image was used to produce a mask of all endosomes and the ratio of EYFP/STAR400 signal was calculated in each endosome (*n* > 150 endosomes for each pH value), averaged for each pH value, and their log_10_ values plotted. Log_10_ EYFP/STAR400 values were fit to a linear equation (*R* ^2^ = 0.99). **d** Confocal and STED images of SRpHi4-loaded CV-1 cells are presented. *Arrows* point to individual endosomes that could only be resolved by STED. Additionally, a ratiometric image was calculated from the STED data and displays the calculated pH of each endosome. **e** CV-1 cells were pulsed with the SRpHi1 or SRpHi4 probes, followed by imaging or a further 60 min probe-free chase where indicated. Ratiometric images were produced as outlined in **c** and the average pH of each endosome calculated. For the top three histograms, the total number of endosomes falling into each 0.5 pH unit bin is displayed. Additionally, the average endosomal pH for each treatment group is presented at the bottom. *Error bars* represent standard deviation from the mean; * represents a *p*-value of <0.05 calculated by a 2-tailed Student’s *t*-test. *Scale bars*: 1 µm

SRpHi probes 1–3 were targeted to the endocytic machinery by the short peptide TAT. As shown in Fig. 2, TAT directed internalization of the SRpHi probes through early and late endosomes to the lysosome. Although useful for bulk cargo flow through the endocytic pathway, TAT does not allow for the study of more specialized intracellular movements such as recycling. Fortunately, the design of the SRpHi probes is modular and they can be easily targeted to other pathways. As a proof-of-principle experiment, SRpHi4 was developed, in which the short TAT sequence was replaced with the mature epidermal growth factor (EGF) protein (Fig. 1a). A 34.4 kDa EYFP-EGF fusion protein was purified (Fig. 6a) and labeled with STAR400, a shorter Stokes-shift dye that is better suited to imaging on commercially available microscopes. Figure 6b displays the rapid uptake of SRpHi4 into early endosomes, with >30% of SRpHi4-containing endosomes colocalizing with EEA1 after 10 min of loading. This is similar to what was seen with the TAT fusion SRpHi2 protein (Fig. 2c). In vivo standard curves were determined for this probe (Fig. 6c) and STED imaging was performed. Once again, STED imaging resolved a number of sub-resolution endosomes and their individual pH values could be determined (Fig. 6d). Finally, we sought to characterize the trafficking differences between our SRpHi1 and SRpHi4 probes that use the TAT peptide and EGF to initiate internalization, respectively. Figure 6e shows that SRpHi4 is found in endosomes with a significantly higher pH than SRpHi1 after a 10-min pulse with each probe (pH = 6.0 vs. 5.7, *p* < 0.05 by Student’s *t*-test). This proof-of-concept experiment demonstrates the usefulness of the SRpHi probes for evaluating pH levels in super-resolved vesicles and comparing them between different cargos. The presence of SRpHi1 (which is internalized via the TAT cell-penetrating peptide) in more acidic endosomes most likely indicates a faster internalization relative to SRpHi4 (in which internalization is facilitated by EFG ligand binding to a cell surface receptor), or a faster progression from early to late endosomes. Consistent with the TAT peptide’s previously described tendency for lysosomal targeting^18^, we found that SRpHi1 pulses followed by a 60-min probe-free chase resulted in the movement of SRpHi1 to even more acidic compartments (average pH = 5.4, Fig. 6e).

## Discussion

A common difficulty in live-cell imaging of endocytic structures involves resolving individual endosomes in tightly packed regions, and observing the dynamics of tubule outgrowth from early endosomes. Often, overexpression of various proteins that enlarge endosomes is used to study these structures, with a potential impact on the physiological relevance of the experiment^7^. Recently, STED imaging has proven superior to standard widefield and confocal analysis for investigating endosomal dynamics in densely packed regions of the cell and in tubular structures^6, 19^. Figures 3c, d, and 6d demonstrate the advantages of STED imaging in areas of high endosome density. STED also presented clear advantages for imaging tubular endosomal structures (Fig. 3d). Tubular structures were rarely seen, perhaps due to the targeting of most SRpHi probes to a degradative, and not recycling pathway; however, tubules that were present were imaged more clearly by STED (Fig. 3d). Most interestingly, a number of structures that appeared as tubules in confocal images, were found to be individual vesicles when visualized by STED (Fig. 3d). Further investigations that fuse SRpHi probes to ligands that are known to have non-canonical endosomal trafficking properties may be able to address this question. Together, these data affirm the necessity of fluorescence nanoscopy for investigations of endosomal structure and pH dynamics.

The SRpHi probes enable sub-diffraction live-cell imaging of pH changes within endosomes and their associated structures. While they were primarily imaged on home-built STED instrumentation in this study, the fluorophores chosen are also compatible with commercial fluorescence, confocal, and STED systems, making this assay widely adoptable. The large dynamic range of these sensors provides a detection level greater than many currently available pH probes.

However, care must be taken when reaching the lower limits of the pH range of the probe. As shown in Figs. 4 and 6, and Supplementary Fig. 4, when the fluorescent protein signal is weak and approaches the background noise level, the variability of the measured ratio at each endosome can begin to increase. Therefore, pH ranges for all standard curves were chosen where even at the lowest pH value the acid-sensitive fluorophore could still be seen above background. Additionally, standard curves always utilized the acid-stable organic dye as the denominator.

Supplementary Fig. 5 shows the average intensity of all endosomes analyzed at pH 5 to obtain the standard curve displayed in Fig. 4b. Compared to 30 randomly chosen ROIs across all of these images, the average signal within the endosomes is ~9-fold larger (average pixel intensities equal to 161 and 18, respectively). In fact, of the nearly 200 endosomes analyzed only one had an average intensity (38) lower than the highest average intensity found in a background ROI (41). Importantly, the variance of the ratiometric measurements rapidly increases at pH values outside of the ranges shown here. Therefore, we do not recommend imaging outside of the optimal pH ranges for each SRpHi probe (Supplementary Table 1). Also, we were concerned that the increased variance may occur if the signal from small endosomes is not sufficient to exceed the noise floor of detection. Indeed, the smallest endosomes are of the most interest as they are only revealed by super-resolution imaging. Fortunately, we found ratiometric variability cannot be attributed to the size of the endosomes, as the average intensity was not directly proportional to endosome size. Supplementary Fig. 5b shows that there is no relation between endosome size and average intensity (*R* ^2^ = 0.10).

Additional advantages of the SRpHi probes include rapid targeting to the endocytic pathway of all mammalian cells tested here, including: lines derived from human, monkey, and mouse and primary cultures of cortical neurons (data not shown). No transfections or genetic modifications are required for SRpHi use. Finally, uptake of SRpHi probes did not appear to affect normal endosomal trafficking or have any other adverse effects on the cells studied here. Together, this suggests these probes are applicable to a wide range of endosomal pH studies. As changes in pH are essential for many cellular functions related to endocytic trafficking, and endosomal structures lie below the diffraction limit of light, these SRpHi probes have the potential to assist in numerous studies of internalization and intracellular transport.

## Methods

### SRpHi probe construction

SRpHi probes were constructed using standard cloning methods. Complementary DNA encoding EYFP, EGFP, or sepHluorin was cloned in frame into the pGEX-6P-1 vector (GE Healthcare, Munich, Germany), downstream of a GST tag, complementary DNA encoding a TAT peptide (GRKKRRQRRR) and a flexible linker. For SRpHi4, EGF was fused to the C-terminus of EYFP. SRpHi probes were expressed in BL21 *Escherichia coli* cells, induced for 24–48 h with 1 mM IPTG, and isolated on GST-Sepharose beads according to the manufacturer’s directions. GST tags were cleaved using PreScission Protease (GE Healthcare, Munich) and the GST moiety was removed by an additional pass over a GST-sepharose column. Unconjugated NHS-ester containing STAR400, STAR410, or STAR512 dyes (Abberior GmbH, Göttingen) were coupled to SRpHi probes according to the manufacturer’s directions. Various dye concentrations were empirically tested to produce a labeling ratio of ~2:1 (dye:probe). Free dye was removed via size exclusion column (illustra NAP-5, MW cutoff = 5000 Da, GE Healthcare, Munich). SRpHi-containing fractions were combined, dialyzed against PBS and concentrated.

### Cell culture and SRpHi loading

MEF cells and HeLa cells were gifts from local research groups and originally obtained from ATCC (Manassas, VA). African Green Monkey (CV-1) cells were obtained from Life Technologies (Darmstadt). All cell lines were cultured on 18 mm round coverslips for 2–3 days in DMEM supplemented with 10% FBS (Life Technologies, Darmstadt) at 37 °C in 5% CO_2_. SRpHi was loaded by placing the coverslip on 20 μL of SRpHi solution diluted in cell growth media to ~30 µM on parafilm and returning this to the incubator for 10 min. Optionally, membrane-bound, uninternalized SRpHi was removed by three washes with 1 mg/mL heparin (Sigma Aldrich, Munich) in PBS or culture medium. Unbound SRpHi was removed by washing three times (one time if preceded by heparin washes) in cold PBS prior to imaging in pre-warmed PBS or phenol-red-free OptiMEM (Life Technologies, Darmstadt). An additional 60–90 min incubation in SRpHi-free growth medium was included for pulse/chase experiments.

### Fixation and immunolabelling

Standard immunofluorescence staining protocols were followed. Cells immunolabelled against LAMP2 were fixed in 100% methanol cooled to −80 °C. All others were fixed in 3–4% paraformaldehyde (w/v) while on ice. Anti-EEA1 and LAMP2 antibodies were from BD Biosciences (Cat # 610456, lot # 78952, Heidelberg) and Santa-Cruz Biotechnologies (Cat # sc-18822, Lot # H0409, Santa-Cruz, CA) and used at dilutions of 1:1000 and 1:500, respectively. Anti-γ-tubulin antibody was obtained from Sigma Aldrich (Cat #T6557, Munich) and was used at a dilution of 1:2000.

### Colocalization

Colocalization was determined by a custom Matlab (The Mathworks, Ismaning) script. The script identified the center of mass of each vesicle (at least 5 pixels in size) in one color channel and determined its nearest neighbor in the corresponding channel. Centers of mass less than the average endosome diameter in pixels were considered colocalized.

### Immunoblotting

Cell lysates were prepared using standard procedures in a Tween-20 (Sigma Aldrich, Munich) containing cell lysis buffer. Cell lysates were separated by SDS-PAGE and transferred to low-fluorescence PVDF membranes (GE Health Sciences, Munich). Membranes were incubated overnight with antibodies directed against gamma-tubulin (Sigma Aldrich, Munich) and imaged on a custom-built gel dock with appropriate excitation and emission filters.

### Imaging

Widefield imaging was performed on an upright Leica DM6000 B fluorescence microscope (Leica, Mannheim). Confocal and STED imaging were performed on a Leica SP8 confocal (Leica, Mannheim) and custom-built systems^11, 15, 17^ (Supplementary Fig. 3).

### Generation of ratiometric standard curves

MEF or CV-1 cells were pulsed as above with SRpHi for 10 min. Cells were transferred to a citric acid/phosphate buffer of specific pH supplemented with 140 mM KCl and 1 µM nigericin (both Sigma Aldrich, Heidelberg) prior to STED imaging. A custom-written macro in ImageJ/Fiji^20, 21^ was used to calculate the intensity ratio of fluorescent protein to fluorescent dye in each endosome. Briefly, input images (16 bit) were set to identical display levels. A mask of endosomes present in the fluorescence channel was created using an Otsu threshold^22^. Using this mask, the average pixel intensity in each endosome (over 150 for each pH value measured) was calculated for both channels and exported to Microsoft Excel, where the logarithms of the ratios of fluorescent protein to fluorescent dye intensity were calculated and graphed.

### Generation of ratiometric images

A custom-written macro in ImageJ/Fiji^20, 21^ was used to produce ratiometric images. Briefly, endosomes in the fluorescence channel were masked as described above. Only pixels contained within these masks were analyzed further. A 32-bit ratiometric image was obtained by dividing the intensity value of each pixel in the fluorescent protein channel with its corresponding pixel in the fluorescent dye channel. A base 10 log of this image was calculated and the image was rescaled (according to the SRpHi calibration curve) and converted to 8 bit. A custom, linear Look Up Table (LUT) was then applied to the image.

### Statistics

All error bars represent standard error of the mean. Significant differences are indicated by * that represent a *p*-value of less than 0.05 as calculated by a two-tailed Student’s *t*-test. At least three biological replicates were carried out for every experiment. Computational automation allowed for large numbers of endosomes to be evaluated where statistics were needed. Therefore, all *t*-tests had a power >0.80 and were able to account for slight deviations in each population from normal distributions if they exist.

### Code availability

All Matlab scripts and ImageJ macros are available from the corresponding authors upon reasonable request.

### Data availability

All relevant data that support the findings of this study are available from the corresponding authors upon reasonable request.

## Electronic supplementary material

Supplementary Information
