# Supplementary material for: SRpHi ratiometric pH biosensors for super-resolution microscopy
Source: Nat Commun. 2017 Sep 18;8:577. doi: 10.1038/s41467-017-00606-4 (PMC5603529; doi:10.1038/s41467-017-00606-4)
Supplement: Supplementary file 1 — Supplementary Information [file 41467_2017_606_MOESM1_ESM.pdf]

Description of Supplementary Files

File Name: Supplementary Information
Description: Supplementary Figures, Supplementary Table and Supplementary Reference

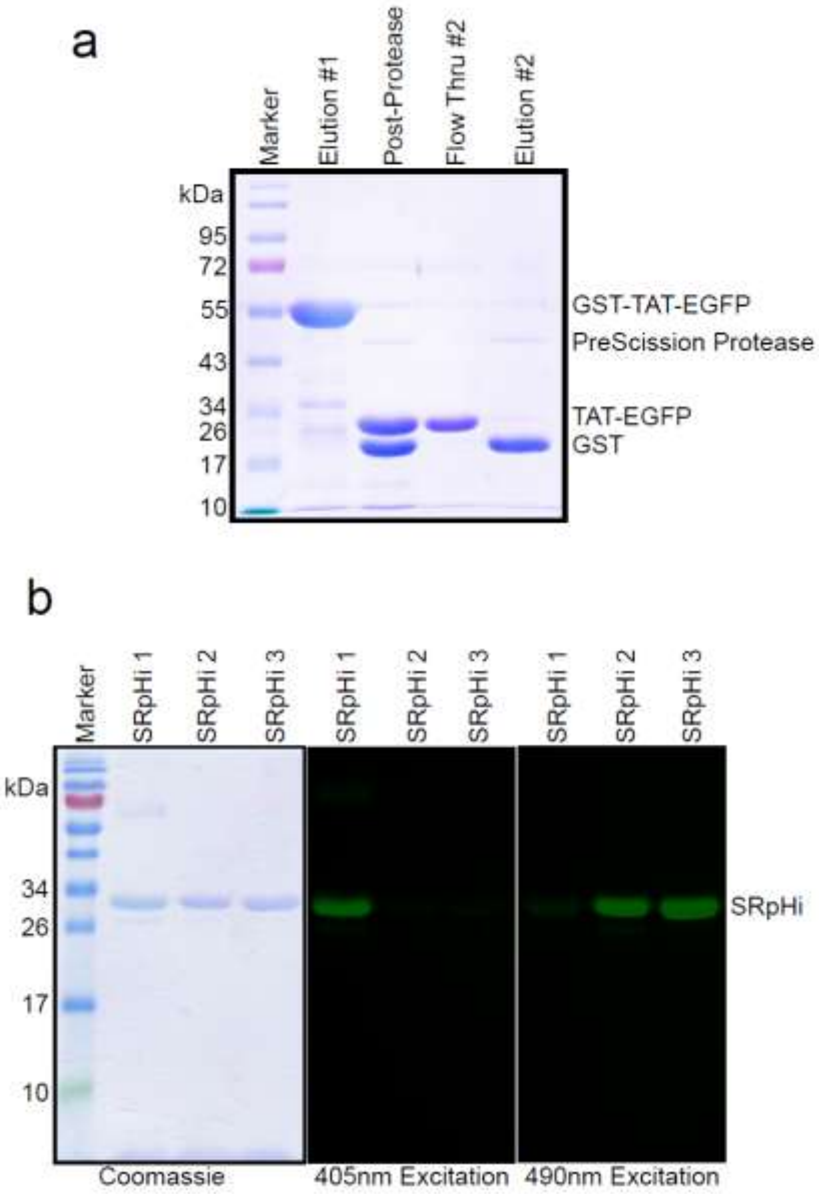

Supplementary Figure 1 | Purification and labeling of SRpHi. (**a**) GST-TAT-EGFP was isolated from bacterial cell lysates by glutathione chromatography (lane 2). The GST moiety was cleaved from TAT-EGFP via PreScission Protease (lane 3) and again passed over a glutathione column. Purified TAT-EGFP was found in the flow-thru (lane 4) while free GST and the GST-tagged protease remained bound in the column. A further elution step was required to obtain these products (lane 5). Protein samples from each step were separated by SDS-PAGE and stained with Coomassie dye. (**b**) Aliquots of SRpHi1, 2, and 3 were separated by SDS-PAGE. Prior to Coomassie staining (left), the presence of STAR410 and STAR512 was assessed by fluorescence gel imaging under the indicated excitation light (right). Native EGFP fluorescence is not seen as the protein has been denatured.

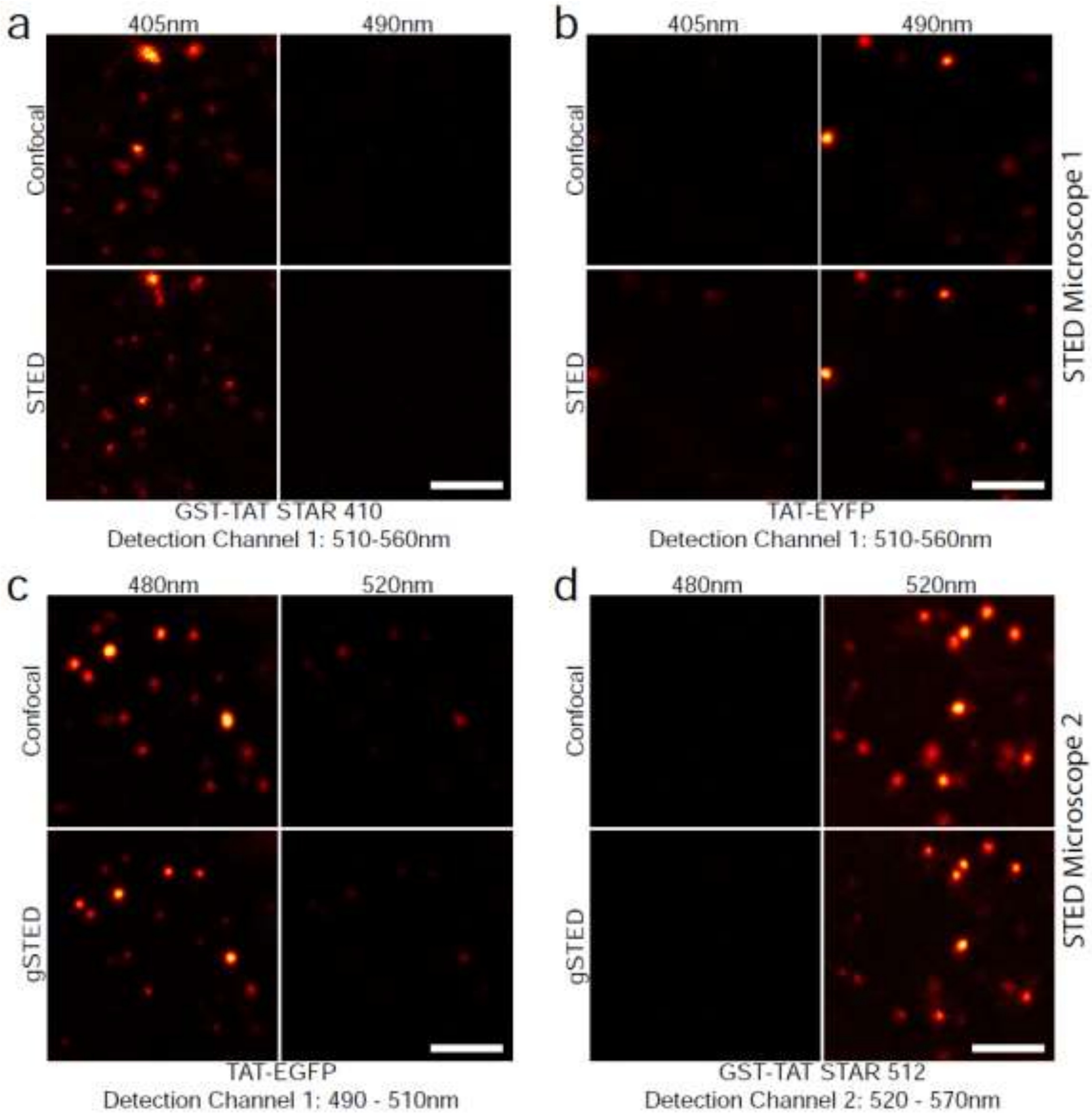

Supplementary Figure 2 | SRpHi probes show negligible cross-talk between channels. (**a-d**) MEF cells were loaded with the indicated constructs containing only a single fluorophore to assess fluorescence bleed-through between channels. Organic dyes (STAR410 and 512) were covalently attached to a purified TAT-GST protein (a,d). TAT-EYFP and TAT-EGFP were purified as described without undergoing fluorophore attachment (b,c). gSTED stands for gated STED (1). Scale bars: 2 μm.

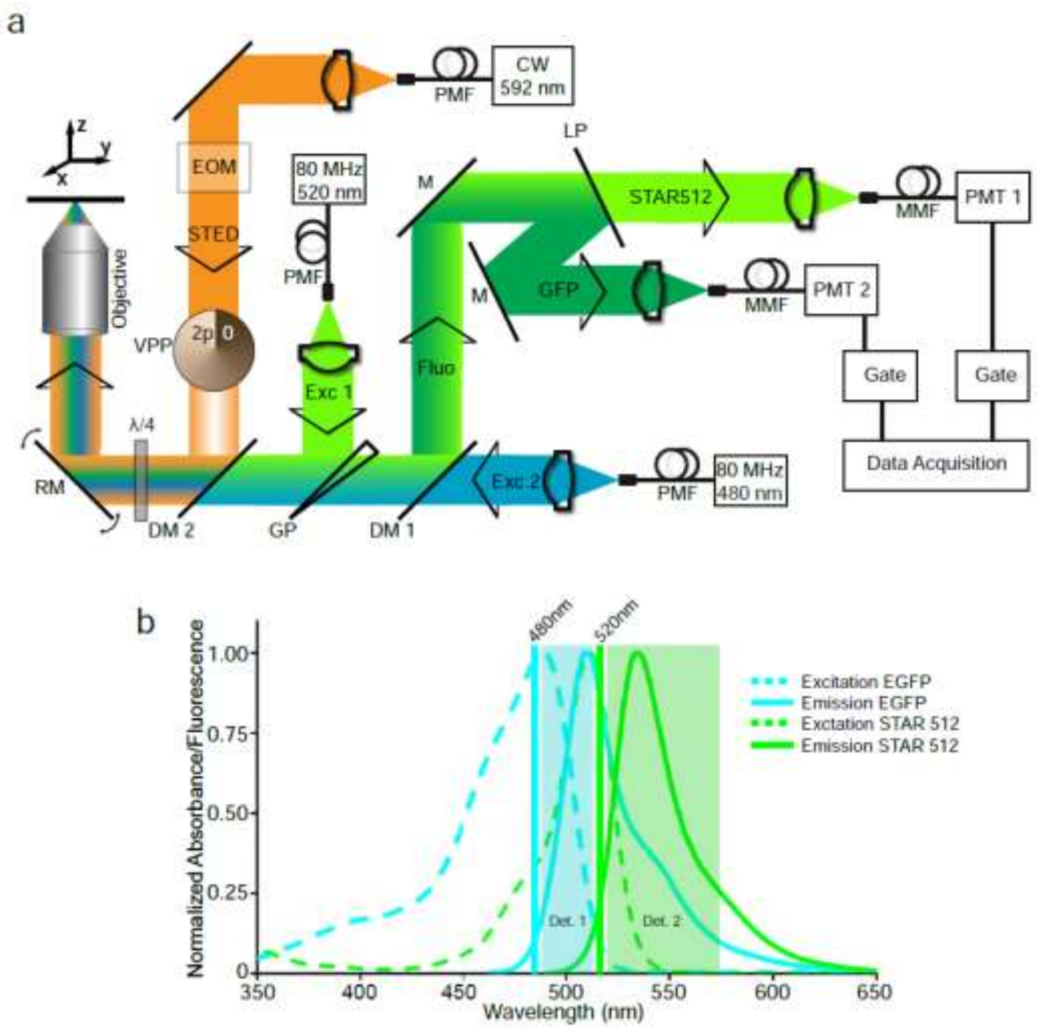

Supplementary Figure 3 | STED image acquisition for SRpHi2 and 3. (**a**) A previous version of this setup has been described[1]. Excitation light from two pulsed diode lasers was merged via a glass plate (GP) and further combined with the continuous-wave STED beam at the dichroic mirror 2 (DM 2). The STED beam was shaped to produce a focal intensity profile with a central minimum by passing through a 2pi vortex phase plate (VPP) and a quarter-wave plate (λ/4). The STED power was controlled via an electro-optic modulator (EOM). A resonance scanning mirror (RM) provided rapid *x*-axis scanning, while a piezo stage was used for scanning of the slow axis. The excitation and STED light were focused into the sample through a 100x/1.4NA oil immersion objective. The sample fluorescence was collected by the objective and back-projected to dichroic mirror 1 (DM 1), where it was separated from the excitation light and passed to a long-pass filter (LP) that allowed separation of EGFP and sepHluorin fluorescence from the STAR512 dye. Both fluorescence channels were passed through multi-mode fibers (MMF) that served as confocal pinholes, on to photon-counting PMTs. The photon counts were passed through a time gate prior to data acquisition. PMF: polarization-maintaining fiber. M: mirror. (**b**) Detection channel 1 (cyan shaded region) and detection channel 2 (green shaded region) overlaid on the absorbance (dotted line) and emission (solid line) spectra of EGFP (cyan) and STAR512 (green).

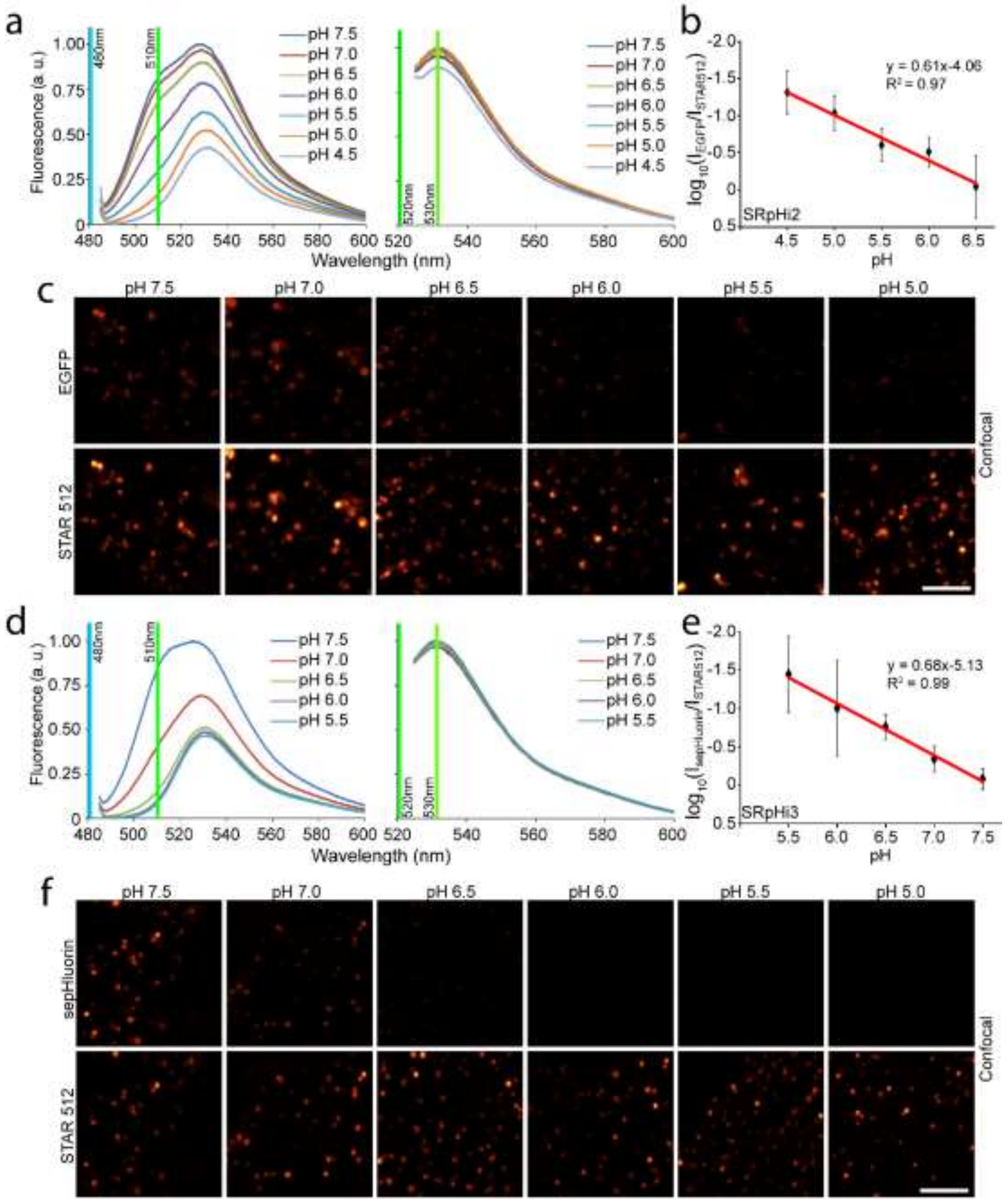

Supplementary Figure 4 | Characterization of SRpHi2 and SRpHi3. (**a**,**d**) Fluorescence emission spectra of SRpHi2 (**a**) and SRpHi3 (**d**) at indicated pH under 480nm or 520 nm excitation. The fluorescence maxima of EGFP (**a**), sepHluorin (**d**) and STAR512 (**a**,**d**) are indicated. (**b**,**e**) EGFP: STAR512 (**b**) or sepHluorin:STAR512 (**e**) fluorescence ratios, as calculated from from live-cell nigericin equilibration experiments. Error bars represent the standard deviation from the mean. Data are from one of 3 independent experiments. (**c**,**f**) MEF cells were incubated with SRpHi2 (**c**) or SRpHi3 (**f**) for 10 minutes prior to imaging. Cells were equilibrated to the indicated pH values by supplementing citric acid/phosphate buffer with nigericin and potassium. Each EGFP or sepHluorin channel is normalized to the corresponding STAR512 image. Scale bars: 2 μm.

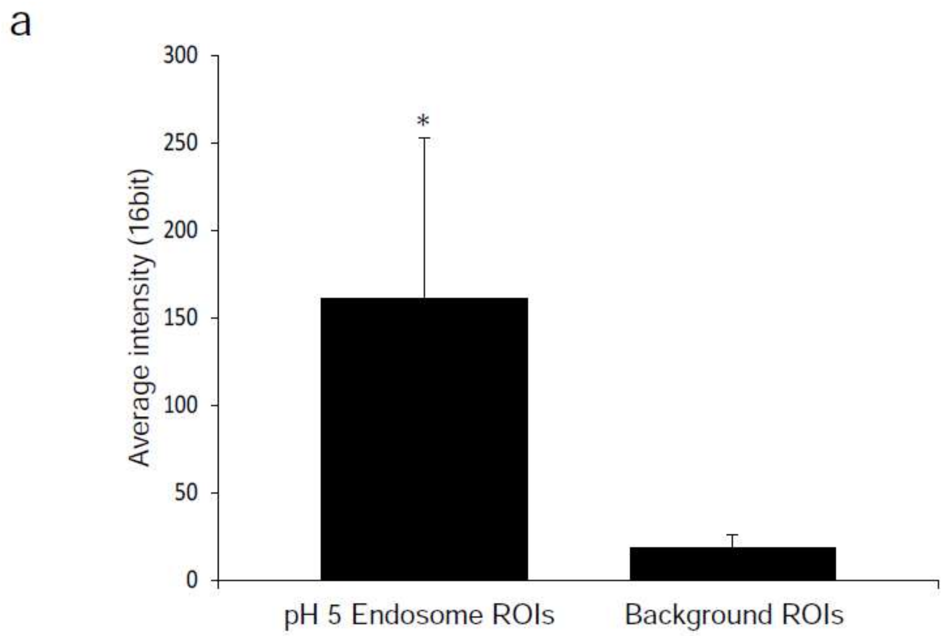

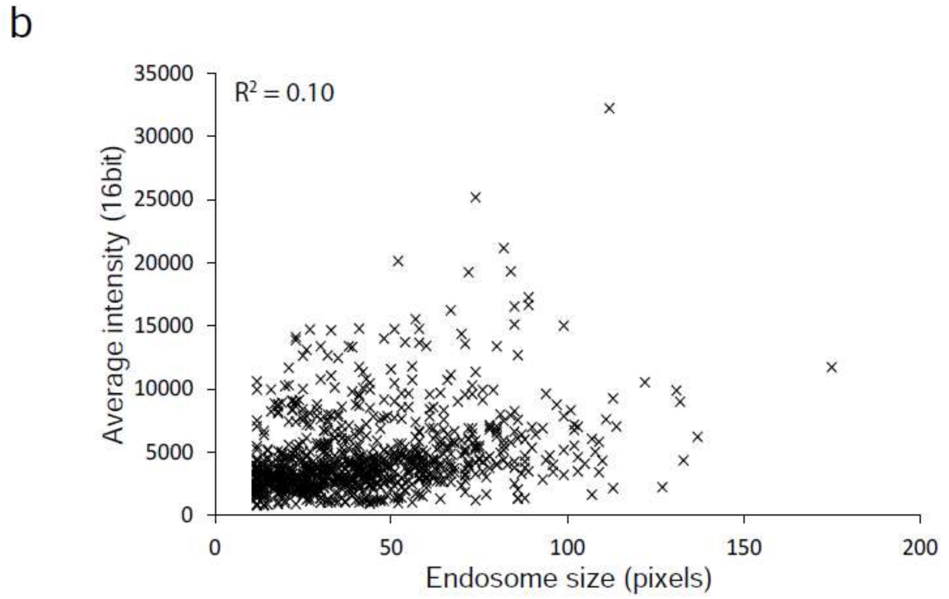

Supplementary Figure 5 | Signal intensity at low pH. (**a**) The average EYFP pixel intensity was calculated for all endosomes (~200) used to generate the pH 5 value of the standard curve in Fig. 4b (main text). Additionally, the average signal intensity in 30 randomly selected ROIs from the same images were also measured and plotted. Error bars represent the standard deviation from the mean and * denotes a significantly enhanced intensity ($p < 0.005$). (**b**) The average pixel intensity and area (number of pixels) was calculated and plotted for each endosome (>900) used to produce the standard curve in Fig. 4b. An R^2 value of 0.10 was calculated from the data. An endosome was required to have an area >10 pixels to be included in the analysis.

	Linear Response Range (pH)	Optimal Endocytic Transition
SRpHi1	7.0 - 5.0	Early/Late Endosome Transition
SRpHi2	6.0 - 4.5	Lysosome Delivery
SRpHi3	7.5 - 6.0	Membrane binding/Internalization/Early Endosome Trafficking
SRpHi4	7.0 - 5.0	Early/Late Endosome Transition

Supplementary Table 1. Recommended pH ranges for SRpHi probes.

Supplementary reference

1. G. Vicidomini, G. Moneron, K. Y. Han *et al.*, *Nat. Methods* **8**, 571-573 (2011).
